# The diagnostic accuracy of a single CEA blood test in detecting colorectal cancer recurrence: Results from the FACS trial

**DOI:** 10.1371/journal.pone.0171810

**Published:** 2017-03-10

**Authors:** Bethany Shinkins, Brian D. Nicholson, John Primrose, Rafael Perera, Timothy James, Sian Pugh, David Mant

**Affiliations:** 1 Test Evaluation Group, Academic Unit of Health Economics, University of Leeds, Leeds, United Kingdom; 2 Nuffield Department of Primary Care Health Sciences, University of Oxford, Oxford, United Kingdom; 3 Academic Unit of Cancer Sciences, University of Southampton, Southampton, United Kingdom; 4 Oxford University Hospitals NHS Foundation Trust, Oxford, United Kingdom; University Hospital Llandough, UNITED KINGDOM

## Abstract

**Objective:**

To evaluate the diagnostic accuracy of a single CEA (carcinoembryonic antigen) blood test in detecting colorectal cancer recurrence.

**Background:**

Patients who have undergone curative resection for primary colorectal cancer are typically followed up with scheduled CEA testing for 5 years. Decisions to investigate further (usually by CT imaging) are based on single test results, reflecting international guidelines.

**Methods:**

A secondary analysis was undertaken of data from the FACS trial (two arms included CEA testing). The composite reference standard applied included CT-CAP imaging, clinical assessment and colonoscopy. Accuracy in detecting recurrence was evaluated in terms of sensitivity, specificity, likelihood ratios, predictive values, time-dependent area under the ROC curves, and operational performance when used prospectively in clinical practice are reported.

**Results:**

Of 582 patients, 104 (17.9%) developed recurrence during the 5 year follow-up period. Applying the recommended threshold of 5μg/L achieves at best 50.0% sensitivity (95% CI: 40.1–59.9%); in prospective use in clinical practice it would lead to 56 missed recurrences (53.8%; 95% CI: 44.2–64.4%) and 89 false alarms (56.7% of 157 patients referred for investigation). Applying a lower threshold of 2.5μg/L would reduce the number of missed recurrences to 36.5% (95% CI: 26.5–46.5%) but would increase the false alarms to 84.2% (924/1097 referred). Some patients are more prone to false alarms than others—at the 5μg/L threshold, the 89 episodes of unnecessary investigation were clustered in 29 individuals.

**Conclusion:**

Our results demonstrated very low sensitivity for CEA, bringing to question whether it could ever be used as an independent triage test. It is not feasible to improve the diagnostic performance of a single test result by reducing the recommended action threshold because of the workload and false alarms generated. Current national and international guidelines merit re-evaluation and options to improve performance, such as making clinical decisions on the basis of CEA trend, should be further assessed.

## Introduction

Colorectal cancer (CRC) is the third most common cancer worldwide, with approximately 40,000 new cases diagnosed each year in the UK [1]. The survival rate for patients diagnosed with CRC has improved notably over the last few decades due to advances in chemotherapy and an increase in the use of hepatic resection surgery [2]. Nonetheless, many patients still go on to experience recurrence and delays in detection may reduce the possibility and effectiveness of surgical intervention [3]. Approximately 85% of recurrences occur within 30 months of surgery, and nearly all occur within 5 years [4]. Consequently, most national and international guidelines recommend that patients who have undergone curative resection for primary colorectal cancer are typically followed up for 5 years with scheduled blood carcino-embryonic antigen (CEA) testing; typically, further investigation with CT imaging is recommended if the CEA test result is above 5μg/L [5, 6].

Earlier this year, our research group completed a Cochrane systematic review of 52 studies exploring the accuracy of a CEA testing in detecting CRC recurrence [7]. We found that very few studies had analysed the accuracy of CEA in a way that represents how single CEA tests would be interpreted in clinical practice (according to guideline recommendations). Many of the studies simply looked at the accuracy of the CEA value measured closest to the time of diagnosis; in others it was unclear as to which CEA value(s) were actually being evaluated. As CEA is actually used as a monitoring test and repeated throughout follow-up, just considering the CEA value closest to diagnosis of recurrence is potentially misleading—for example, it is likely to underestimate the number of false positive results (i.e. results which trigger unnecessary CT and other investigations because the CEA is raised when no recurrence is detectable). A recent audit of the results of CEA follow-up highlighted the scale of this problem in clinical practice, with further imaging investigations triggered by a CEA level above the recommended threshold failing to confirm recurrence in 50% of cases [8].

Since the initiation of our review, the interim findings of the FACS (Follow-up After Colorectal Surgery) trial have been published [9]. This trial is the largest and most up to date randomised trial comparing different follow-up strategies for patients who have undergone curative surgery for primary colorectal cancer. Here we present a secondary analysis reporting the diagnostic accuracy of CEA within this trial. We report analyses consistent with those identified in existing studies (in order to facilitate inclusion in the next review update), but also an additional analysis which demonstrates how CEA would perform prospectively in clinical practice if guideline recommendations are followed.

## Methods

### Study design

The data originates from the FACS (Follow-up After Colorectal Surgery) trial, a 2x2 pragmatic randomised factorial controlled trial comparing minimum post-surgery follow-up of CRC patients with 3–6 monthly CEA tests and 6–12 monthly computerised tomography (CT) imaging [9]. All participants were recruited between January 2003 and August 2009. We analysed the two arms of the trial which required frequent CEA testing (CEA only arm: n = 300, CEA&CT arm: n = 302). The data analysed is available in S1 Dataset.

Ethical approval for the trial was granted by the National Health Service (NHS) South-West Research Ethics Committee. Participants provided their written consent to participate in this study

Of the 602 individuals included in the analysis, 106 had a recurrence within the 5 year follow-up period (17.3%). The relatively low recurrence rate in this study has implications on the precision in which we can estimate sensitivity. Assuming a sensitivity of 60%, 70% or 80%, we can expect 95% confidence intervals of 50.6–69.4%, 61.2–78.8%, or 72.3–87.7% respectively.

### Follow-up schedule

**Fig 1:**
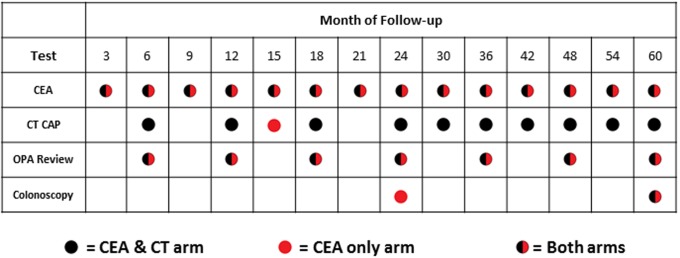
5-year follow-up schedule.

The follow-up schedule in both arms included CEA measurements every 3 months for the first 2 years and then every 6 months for the following 3 years. A composite reference standard was used which consisted of CT CAP, clinical assessment at outpatient appointment (OPA review) and colonoscopy. Those randomised to the CEA&CT arm received more frequent CT CAP (see Fig 1. for details of schedule).

Any CEA value 7μg/L or more above baseline during follow-up was repeated within two weeks. If the repeat CEA test was again >7μg/L, the GP urgently referred the patient for off-protocol investigations for recurrence determined by the local surgeon. If the repeat CEA test was <7μg/L above baseline the patient continued in the study as per protocol.

As a pragmatic open trial, it was not possible to conceal the allocation group from participants or clinicians, and radiologists reporting off protocol scans triggered by a rise in CEA were not blinded to the reason for scanning.

### Technical specifications

Blood collection kits were sent directly to patients, who then attended their own general practice for phlebotomy. Blood samples were sent to the Biochemistry laboratory at the John Radcliffe Hospital laboratory, Oxford. All CEA samples were analysed using a Siemens Centaur XP analyser. Repeat tests were requested by sending a letter and sample kit to the patient asking them to schedule an appointment at their GPs for the repeat test. Imaging studies were reported by a consultant radiologist.

All CT imaging was conducted on the highest quality equipment available in the collaborating centres. MRI scanning was an acceptable alternative to CT if performed in a study centre.

### Statistical analyses

Two alternative methods for evaluating the accuracy of CEA were identified in our Cochrane review—a ‘final CEA value’ analysis and an ‘any rise’ analysis [10]. For the ‘final CEA value’ analysis, the accuracy of the CEA measurement taken closest to the time that recurrence for predicting recurrence is reported. For patients that do not experience recurrence, the CEA measurement taken at the end of follow-up is evaluated instead. Alternatively, as CEA is being monitored and CEA measurements are interpreted prospectively, the ‘any rise’ analysis involved looking across all CEA measurements available for any rise in CEA above a given threshold. We replicate both of these analytic approaches here.

Receiver Operating Characteristic (ROC) curves are produced alongside 95% bootstrap confidence intervals to explore how accuracy varies with threshold using the pROC package in R [11]. The area under the curve is also presented. The 2 x 2 data underpinning these plots is available on request for meta-analysis purposes. Given that censoring is present in the dataset under analysis, we also produced time-dependent ROC curves [12] and report the associated AUCs at years 1–5. Sensitivity and specificity, likelihood ratios, and predictive values (along with their respective 95% confidence intervals) are summarised for the most commonly recommended and implemented threshold of 5μg/L.

An operational analysis of the likely impact of CEA testing if used prospectively in clinical practice was also conducted, hypothetically applying different absolute thresholds (from 2.5 to 10 μg/L) to trigger further investigation on the basis of the result of each individual test done during the follow-up period. In relation to the CEA test scheduled at each time point, two outcomes are reported: 1) the proportion of recurrences that would have been missed and not referred for further investigation because the CEA level was below the threshold; 2) the proportion of patients with a CEA level above the threshold who were subject to a false alarm (i.e. an unnecessary referral for further investigation of a patient who did not experience recurrence throughout the whole follow-up period). The data for the 7.5μg/L and 10μg/L threshold are less robust than for 5μg/L and 2.5μg/L thresholds as they were above the action threshold applied in the FACS trial and therefore influenced more by work-up bias; they are reported in S2 and S3 Tables for completeness.

The R code for the analyses reported is available in S1 Code File.

## Results

The patient recruitment process for FACS and the reasons for any exclusions can be found in S1 Fig. Of the 582 patients undergoing CEA follow-up, 104 (17.9%; 95% CI: 15.0% to 21.2%) developed a recurrence during the 5-year follow-up period. As expected, the primary cancer was more advanced among those who went on to experience recurrence. Other than this, there were no notable differences between those who experienced recurrence and those who did not (see S1 Table).

### Diagnostic accuracy

**Fig 2:**
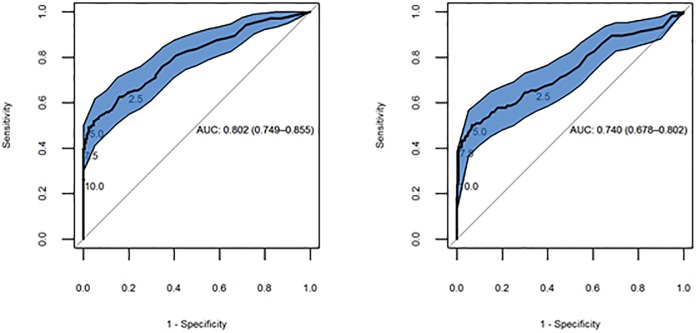
ROC curves depicting the accuracy of the final CEA value (left) and any rise above threshold (right). The sensitivities and specificities achieved at thresholds of 2.5, 5, 7.5 and 10μg/L are highlighted.

As described in the methods, we estimated accuracy in relation to: 1) any rise across all CEA measurements done prior to diagnosis; 2) the final CEA measurement done before the diagnosis. Fig 2 depicts accuracy for these two analyses across all thresholds; the area under the ROC curve (AUC) is slightly better when just the final CEA value taken is analysed compared to looking across all measurements (final CEA value AUC = 0.80, 95% CI: 0.75–0.86); all CEA measurements AUC = 0.74, 95% CI: 0.68–0.80).

**Fig 3:**
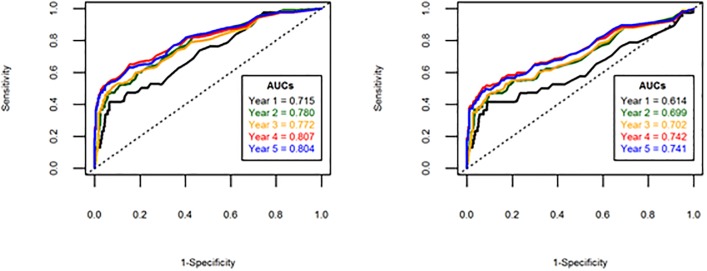
Time-dependent ROC curves depicting the accuracy of the final CEA value (left) and any rise above threshold (right).

Due to the censored nature of the CEA data, time-dependent ROC curves were also produced for years 1–5 (Fig 3). In both analyses, it is evident from the reported area under the ROC curves that the accuracy of CEA is notably poorer for patients who recur in the first year of follow-up compared to subsequent years.

Table 1 presents the sensitivity, specificity, likelihood ratios, and predictive values for both analyses at the commonly recommended threshold of 5μg/L. Including only the final CEA test results in a slightly lower estimate of sensitivity and negative likelihood ratio but a more favourable estimate of all the other diagnostic performance parameters. However, both analyses are retrospective and therefore provide a potentially misleading picture of how CEA performs operationally when used prospectively in clinical practice.

**Table 1:** Comparison of the diagnostic performance in detecting recurrence of a rise in blood CEA level above a threshold of 5μg/L in relation to i) all CEA measurements taken prior to diagnosis of recurrence and ii) the final CEA measurement taken prior to diagnosis of recurrence.

	Final CEA measurement (95% CI)	All CEA measurements (95% CI)
**Sensitivity**	49.0% (39.2–59.0%)	50.0% (40.1–59.9%)
**Specificity**	97.5% (95.5–98.6%)	93.3% (90.6–95.3%)
**Positive predictive value**	81.0% (68.7–89.4%)	61.9% (50.6–72.1%)
**Negative predictive value**	89.8% (86.8–92.2%)	89.6% (86.5–92.0%)
**Positive likelihood ratio**	19.5 (10.8–35.3)	7.5 (5.1–11.0)
**Negative likelihood ratio**	0.52 (0.43–0.63)	0.54 (0.44–0.65)

### Operational performance

Table 2 shows the estimated operational performance of CEA monitoring in clinical practice when two different thresholds for initiating further investigation are applied prospectively: the current standard threshold of 5μg/L and a potentially more sensitive threshold of 2.5μg/L.

**Table 2:** Estimated operational performance of CEA in clinical practice at currently recommended intervals if further investigation is triggered by thresholds of 2.5 and 5 μg/L.

Time of test		CEA tests	Recurrences	5μg/L action threshold		2.5 μg/L action threshold	
				Missed cases	False alarms	Missed cases	False alarms
*Year*	*Month*	*n*	*n*	*n (%)*	*n/N (%)*	*n (%)*	*n/N (%)*
**Year 1**	3	563	15	9	3/15	7	63/88
	6	542	17	10	6/16	10	68/92
	9	530	7	3	2/9	2	68/87
	12	519	12	7	8/15	3	66/86
	All	2154	51	29 (56.9%)	19/55 (34.5%)	22 (43.1%)	265/353 (75.1%)
**Year 2**	15	500	7	3	2/8	1	62/76
	18	493	7	4	9/12	2	67/80
	21	482	1	0	6/7	0	64/70
	24	477	11	7	8/12	5	65/78
	All	1952	26	14 (53.8%)	25/39 (64.1%)	8 (30.8%)	258/304 (84.9%)
**Year 3**	30	455	6	4	7/10	4	67/76
	36	444	7	1	9/16	0	68/80
	All	899	13	5 (38.5%)	16/26 (61.5%)	4 (30.8%)	135/156 (86.5%)
**Year 4**	42	427	6	2	6/11	0	71/81
	48	408	5	3	7/9	2	69/74
	All	835	11	5 (45.5%)	13/20 (65.0%)	2 (18.2%)	140/155 (90.3%)
**Year 5**	54	395	2	2	12/12	2	65/66
	60	374	1	1	4/5	0	61/63
	All	769	3	3 (100%)	16/17 (94.1%)	2 (66.7%)	126/129 (97.7%)
**All years**		**6609**	**104**	**56 (53.8%)**	**89/157 (56.7%)**	**38 (36.5%)**	**924/1097 (84.2%)**

The number of recurrences diagnosed by the composite gold standard (and therefore potentially identifiable by a rise in CEA) in each testing period falls from a mean of 12.8 in year 1 (2.2% of patients at risk) to 1.5 in year 5 (0.31% of patients at risk), despite the reduction in frequency of testing from 3 to 6 monthly after year 2. Overall, CEA correctly identifies the need for further investigation in just under half the cases of recurrence applying a threshold of 5μg/L (46.2%, 95% CI: 36.6–55.8) and just under two-thirds with a threshold of 2.5μg/L (63.5%, 95%CI: 54.2–72.8). There is no clear trend in the proportion of missed cases over time. If the threshold is increased, the overall number of missed cases over the 5 year follow-up period increases to 62.5% at 7.5μg/L and 75.0% at 10μg/L (S2 and S3 Tables).

### Unnecessary investigations

Table 2 also shows the cost of reducing the threshold from 5 to 2.5μg/L in terms of investigative workload and false alarms. At 5μg/L, only 157 of 6609 (2.4%) of CEA tests trigger further investigation compared to 1097 (16.6%) at 2.5μg/L. The number of false alarms increases from about 1 in 2 (56.7%) at 5μg/L to 5 in 6 (84.2%) at 2.5μg/L. The proportion of false alarms also increases over time with both thresholds—from 34.5 to 94.5% at 5μg/L and from 75.1 to 97.7% at 2.5μg/L in years 1 and 5 respectively.

Some patients are more prone to false alarms than others. For example, the 89 unnecessary investigations triggered at a threshold of 5μg/L were clustered in 29 individuals, 15 of whom (51.7%) would have more than one false alarm, 8 more than 5 false alarms. At a threshold of 2.5μg/L, the 924 false alarms are clustered in 156 people, of whom 114 (73.0%) would have more than one false alarm and 69 more than 5 false alarms. The estimated maximum number of false alarms in one patient over a 5 year period are 10 at 5μg/L and 14 at 2.5μg/L. Full details are given in S4 Table.

S2 and S3 Tables show that unnecessary investigations are a much smaller problem with the higher thresholds of 7.5 and 10μg/L, with only 4 and 2 false alarms generated respectively, with no patients suffering more than one false alarm.

## Discussion

### Main findings

CEA must not be used alone as a means of monitoring for colorectal cancer recurrence. Whatever threshold is applied, a significant number of patients suffer recurrence without a rise in CEA levels. At the ASCO recommended threshold of 5μg/L, about half the recurrences will be missed. This underlines the importance of combining CEA with scheduled imaging, as recommended in most national guidelines. In the comparative analysis of the FACS trial, when combined with a single CT-CAP scan at 12–18 months, CEA follow-up performed as well in detecting treatable recurrence as 6-monthly CT-CAP imaging [9].

Although the sensitivity of CEA testing can be increased by reducing the threshold to 2.5μg/L (approximately the threshold recommended as optimal by Tan et al [13]), the cost is high. A 7-fold increase in imaging workload is not sustainable in many health economies (such as the UK NHS) and the very high rate of false alarms (5 out of 6 referrals for further investigation) is unlikely to be acceptable to patients. Importantly, some people will suffer recurrent false alarms.

### Consistency with existing evidence

The modest sensitivity of CEA at the recommended 5μg/L threshold is well documented—in our Cochrane review, the pooled sensitivity based on 23 studies was higher than estimated by our own data (71%, 95% CI: 64–76%) but nearly a third of cases would still be missed [10]. We have criticised the meta-analysis methods used by Tan et al in their 2009 systematic review, but for comparison they reported a sensitivity of 63% at a threshold of 5μg/L and 84% at a threshold of 2.2μg/L [13]. Our reported specificity is also consistent with previous studies although operational performance (i.e. imaging workload and false alarms) will depend on testing interval and prevalence of recurrence in the population being followed-up.

### Strengths and limitations

The main strengths of the FACS trial data in this context is that CEA testing was centrally managed with high compliance with scheduled testing and all analyses being done in one laboratory with consistent quality control. The main strength of this analysis compared to previous diagnostic accuracy studies of CEA in detecting recurrence of which we are aware, is that we modelled the operational performance of CEA when used prospectively in clinical practice, rather than simply looking retrospectively at sensitivity and specificity in relation to a series of tests.

The main limitation of the data is that we do not have a reference standard at all time points. We do not know the precise time when a recurrence would have been detectable by our gold standard. Our estimate of unnecessary referrals is likely to be an underestimate as it only includes patients who did not experience recurrence throughout the whole follow-up period. However, the length of follow-up and within-trial surveillance means that we are unlikely to have missed cases of recurrence. Even if we had been able to apply the gold standard at every time-point, there may be a lead-time between detectability of recurrence by CEA and by imaging.

The other important limitation is work-up bias. Patients in the FACS with a CEA>7μg/L above their personal baseline were referred for further investigation. This relatively high threshold means that the analyses of diagnostic accuracy at the thresholds of 2.5μg/L and 7.5μg/L are largely not subject to bias but the estimates of operational performance at 10μg/L reported in the S2 and S3 Tables are less robust.

Finally, the analysis we report here (and most previous studies of which we are aware) assess the diagnostic performance of CEA in detecting any recurrence. We did not have a sufficient number of cases of recurrence to stratify the analysis by the treatability of recurrence.

### Implications for clinical practice and research

CEA has three great advantages over other forms of follow-up: it is relatively inexpensive, can be done in a community setting, and does not expose the patient to radiation. It can therefore be done more frequently than other tests and has the potential to provide important lead-time in detecting recurrence (about 3 months in the FACS trial). However, our data underlines that it must not be used alone as a triage test because of its low sensitivity.

Sensitivity can clearly be increased by reducing the threshold but, as stated above, the impact on workload and high false alarm rate make that a very unattractive solution. However, it is probably time to stop making decisions to investigate further on the basis of a single test result when a series of tests have been done. About 40 years ago Minton et al argued convincingly that the diagnostic accuracy of blood CEA testing in detecting colorectal cancer recurrence would be improved by taking account of trend over time [14] and others have since provided strong supportive evidence [15, 16]. We have already shown on the basis of FACS data that much better diagnostic performance, including better sensitivity, can be achieved by basing the decision to investigate further on the regression co-efficient derived from a series of tests [17].

The main implication of our data for further research is that traditional methods of assessing and reporting the diagnostic accuracy of a single test provide an inadequate guide to the clinical performance of a test which is actually used as a monitoring tool in practice. Research into the accuracy of monitoring tests needs to be designed to assess performance at each individual time point and to take account of the added information provided by sequential testing. Further research into the value of CEA as a monitoring test needs to focus on the detection of recurrence at an optimal stage for treatment.

## Supporting information

S1 Fig Flow-chart of patients allocated to CEA testing within the FACS cohort to show origin of the data analysed here. (DOCX)

S1 Table Patient characteristics for those who did and did not experience recurrence during the 5yr follow-up period. (DOCX)

S2 Table Performance of CEA throughout the follow-up period if a threshold of 7.5μg/L had been implemented. (DOCX)

S3 Table Performance of CEA throughout the follow-up period if a threshold of 10μg/L had been implemented. (DOCX)

S4 Table Clustering of false alarms: Number of times an individual patient who never recurrence would have a CEA measurements over the threshold during the 5 year follow-up period (n = 478). (DOCX)

S1 Dataset Raw CEA data from the FACS trials. Includes CEA measurements and the time from baseline at which the measurements were taken.
(CSV)

S1 Code File R code for reported analyses. (R)
