# Supplementary material for: The diagnostic accuracy of a single CEA blood test in detecting colorectal cancer recurrence: Results from the FACS trial
Source: PLoS One. 2017 Mar 10;12(3):e0171810. doi: 10.1371/journal.pone.0171810 (PMC5345794; doi:10.1371/journal.pone.0171810)
Supplement: S1 Fig — (DOCX) [file pone.0171810.s001.docx]

**S1 Fig. Flow-chart of patients allocated to CEA testing within the FACS cohort to show origin of the data analysed here**

**Randomised to CEA-only arm**
(n=300)

**Randomised to CEA&CT arm**
(n=302)

**Recruits**

(n=602)

**Exclusions** (n=20)

- Postoperative deaths (n=10)
- Withdrawn (n=9)
- Lost to follow up (n=1)

**Followed up**

(n=582)

**Non-recurrence**

(n=478)

**Recurrence** (n=104)
