# Supplementary material for: The diagnostic accuracy of a single CEA blood test in detecting colorectal cancer recurrence: Results from the FACS trial
Source: PLoS One. 2017 Mar 10;12(3):e0171810. doi: 10.1371/journal.pone.0171810 (PMC5345794; doi:10.1371/journal.pone.0171810)
Supplement: S1 Table — (DOCX) [file pone.0171810.s002.docx]

**S2 Table. Patient characteristics for those who did and did not experience recurrence during the 5yr follow-up period**

|  | No CRC Recurrence N(%)  (N=478) | CRC Recurrence N(%)  (N=104) |
| --- | --- | --- |
| Age (years) |  |  |
| <65 | 143 (30%) | 42 (40%) |
| 65-79 | 286 (60%) | 53 (51%) |
| 80 + | 49 (10%) | 9 (9%) |
|  |  |  |
| Sex |  |  |
| Male | 288 (60%) | 69 (%) |
| Female | 190 (40%) | 35 (%) |
|  |  |  |
| Smoking Status |  |  |
| Never Smoked | 195 (41%) | 37 (36%) |
| Ex-Smoker | 248 (52%) | 49 (47%) |
| Current Smoker | 25 (5%) | 11 (11%) |
| Missing | 10 (2%) | 7 (7%) |
|  |  |  |
| Comorbidity at Baseline |  |  |
| No | 340 (71%) | 77 (74%) |
| Yes | 138 (29%) | 27 (26%) |
|  |  |  |
| Dukes Stage of Primary Cancer |  |  |
| A | 100 (21%) | 10 (10%) |
| B | 236 (49%) | 46 (44%) |
| C | 121 (25%) | 45 (43%) |
| Missing | 21 (4%) | 3 (3%) |
|  |  |  |
| T-Stage of Primary Cancer |  |  |
| 0 | 11 (2%) | 0 (0%) |
| 1 | 38 (8%) | 3 (3%) |
| 2 | 86 (18%) | 10 (10%) |
| 3 | 273 (57%) | 65 (63%) |
| 4 | 48 (10%) | 19 (18%) |
| Missing | 22 (5%) | 7 (7%) |
|  |  |  |
| N-Stage of Primary Cancer |  |  |
| 0 | 353 (74%) | 57 (55%) |
| 1 | 83 (17%) | 25 (24%) |
| 2 | 28 (6%) | 18 (17%) |
| 3 | 0 (0%) | 1 (1%) |
| Missing | 14 (3%) | 3 (3%) |
