# Supplementary material for: The diagnostic accuracy of a single CEA blood test in detecting colorectal cancer recurrence: Results from the FACS trial
Source: PLoS One. 2017 Mar 10;12(3):e0171810. doi: 10.1371/journal.pone.0171810 (PMC5345794; doi:10.1371/journal.pone.0171810)
Supplement: S2 Table — (DOCX) [file pone.0171810.s003.docx]

**S2 Table. Performance of CEA throughout the follow-up period if a threshold of 7.5µg/L had been implemented.**

| Month of follow-up | Number clinically diagnosed with recurrence before next measurement | Referrals for raised CEA > 7.5 | Cases of recurrence referred for further investigation | Cases of recurrence missed | Definite false alarms (patients referred when recurrence definitely not present)* |
| --- | --- | --- | --- | --- | --- |
| 3 | 15 | 8 | 4 | 11 | 1 |
| 6 | 17 | 8 | 7 | 10 | 0 |
| 9 | 7 | 5 | 3 | 4 | 0 |
| 12 | 12 | 6 | 5 | 7 | 0 |
| Year One | **51** | **27** | **19** | **32** | **1** |
| 15 | 7 | 4 | 4 | 3 | 0 |
| 18 | 7 | 3 | 3 | 4 | 0 |
| 21 | 1 | 1 | 0 | 1 | 1 |
| 24 | 11 | 2 | 2 | 9 | 0 |
| Year Two | **26** | **10** | **9** | **17** | **1** |
| 30 | 6 | 2 | 1 | 5 | 1 |
| 36 | 7 | 6 | 5 | 2 | 1 |
| Year Three | **13** | **8** | **6** | **7** | **2** |
| 42 | 6 | 2 | 2 | 4 | 0 |
| 48 | 5 | 2 | 2 | 3 | 0 |
| Year Four | **11** | **4** | **4** | **7** | **0** |
| 54 | 2 | 0 | 0 | 2 | 0 |
| 60 | 1 | 1 | 1 | 0 | 0 |
| Year Five | **3** | **1** | **1** | **2** | **0** |
| Total | **104** | **50** | **39** | **65** | **4** |

*Only includes patients who do not experience recurrence during the whole follow-up period. Note: patients can be referred more than once.
