# Supplementary material for: The diagnostic accuracy of a single CEA blood test in detecting colorectal cancer recurrence: Results from the FACS trial
Source: PLoS One. 2017 Mar 10;12(3):e0171810. doi: 10.1371/journal.pone.0171810 (PMC5345794; doi:10.1371/journal.pone.0171810)
Supplement: S4 Table — (DOCX) [file pone.0171810.s005.docx]

**S4 Table. Clustering of false alarms: number of times an individual patient who never recurrence would have a CEA measurements over the threshold during the 5 year follow-up period (n=478)**

|  | **Number of CEA measurements over the threshold** | | | | | | | | | | | | | | |
| --- | --- | --- | --- | --- | --- | --- | --- | --- | --- | --- | --- | --- | --- | --- | --- |
|  | **1** | **2** | **3** | **4** | **5** | **6** | **7** | **8** | **9** | **10** | **11** | **12** | **13** | **14** | **Total** |
| **2.5µg/L** | 42 | 18 | 13 | 7 | 7 | 3 | 7 | 5 | 9 | 4 | 10 | 8 | 11 | 12 | 156 |
| **5µg/L** | 14 | 5 | 1 | 1 | 2 | 2 | 1 | 0 | 1 | 2 | 0 | 0 | 0 | 0 | 29 |
